# Glycemic Gap as a Useful Surrogate Marker for Glucose Variability and Progression of Diabetic Retinopathy

**DOI:** 10.3390/jpm11080799

**Published:** 2021-08-16

**Authors:** Shi-Chue Hsing, Chin Lin, Jiann-Torng Chen, Yi-Hao Chen, Wen-Hui Fang

**Affiliations:** 1 National Defense Medical Center, Department of Internal Medicine, Tri-Service General Hospital, Taipei 11490, Taiwan; lars0121@gmail.com; 2 National Defense Medical Center, Graduate Institute of Life Sciences, Taipei 11490, Taiwan; xup6fup0629@gmail.com; 3 National Defense Medical Center, School of Medicine, Taipei 11490, Taiwan; 4 National Defense Medical Center, School of Public Health, Taipei 11490, Taiwan; 5 National Defense Medical Center, Department of Ophthalmology, Tri-Service General Hospital, Taipei 11490, Taiwan; jt66chen@gmail.com (J.-T.C.); doc30879@mail.ndmctsgh.edu.tw (Y.-H.C.); 6 National Defense Medical Center, Department of Family and Community Medicine, Tri-Service General Hospital, No.161, Min-Chun E. Rd., Sec. 6, Neihu, Taipei 11490, Taiwan

**Keywords:** diabetic retinopathy, type 2 diabetes, progression, glycemic gap, glucose variability

## Abstract

(1) Background: Recent studies have reported that the glucose variability (GV), irrespective of glycosylated hemoglobin (HbA1c), could be an additional risk factor for the development of diabetic retinopathy (DR). However, measurements for GV, such as continuous glucose monitoring (CGM) and fasting plasma glucose (FPG) variability, are expensive and time consuming. (2) Methods: This present study aims to explore the correlation between the glycemic gap as a measurement of GV, and DR. In total, 2565 patients were included in this study. We evaluated the effect of the different types of glycemic gaps on DR progression. (3) Results: We found that the area under the curve (AUC) values of both the glycemic gap and negative glycemic gap showed an association with DR progression. (4) Conclusions: On eliminating the possible influences of chronic blood glucose controls, the results show that GV has deleterious effects that are associated with the progression of DR. The glycemic gap is a simple measurement of GV, and the predictive value of the negative glycemic gap in DR progression shows that GV and treatment-related hypoglycemia may cause the development of DR. Individual treatment goals with a reasonable HbA1c and minimal glucose fluctuations may help in preventing DR.

## 1. Introduction

Diabetic retinopathy (DR) has been identified as one of the major microvascular complications of diabetes mellitus (DM). The common risk factors for DR include poor glycemic control, disease duration, and systolic hypertension. Glycosylated hemoglobin (HbA1c), which indicates an average glucose level over the previous 2–3 months, is the strongest marker of glycemic control [1,2]. However, studies have found a U-shaped association between the HbA1c and mortality in patients, and tight glucose control did not improve healthcare outcomes [3,4]. These findings have suggested that aside from HbA1c, other factors of glycemic control may be related to development of diabetes-related complications.

Recently, studies reported that glucose variability (GV), irrespective of HbA1c, could be an additional risk factor for the development of DR. These measurements for short- and long-term GV, including HbA1c variability, continuous glucose monitoring (CGM), and fasting plasma glucose (FPG) variability were associated with the risk of DR in patients with type 2 DM [5,6,7,8].

High HbA1c standard deviations were reported as a predictor for DR development in two longitudinal studies with a large sample size among Asians with type 2 DM and long follow-up period [9,10]. In addition, the HbA1c standard deviation may play a greater role in DR development than the mean HbA1c when the HbA1c variation magnitude is wide. However, a wide standard deviation only indicates that the values are spread out over a wider range without directionality. We could not explain that the effect was caused by hyperglycemia due to poor control or iatrogenic hypoglycemia due to an intensive HbA1c treatment goal.

In a previous study, the glycemic gap, which is calculated from the glucose level minus the HbA1c-derived average glucose, was used to evaluate stress-induced hyperglycemia (SIH). The results suggested that the glycemic gap was associated with disease severity and adverse clinical outcomes in diabetic patients with liver abscesses, pneumonia, acute myocardial infarction, acute ischemic stroke, chronic obstructive lung disease, necrotizing fasciitis, and in-hospital cardiac arrest [11,12,13].

We hypothesized that GV is associated with an increased risk of developing DR in type 2 DM patients, and that the glycemic gap could be a simple measurement for GV. The aims of the present study were to explore the correlation between the glycemic gap as a measurement for GV, and DR, and to evaluate the effect of a negative/positive magnitude of GV in the development of DR. In addition, we sought to justify the use of the glycemic gap as a tool to reflect individual patterns of glycemic control beyond the HbA1c level.

## 2. Materials and Methods

### 2.1. Population

**Figure 1:**
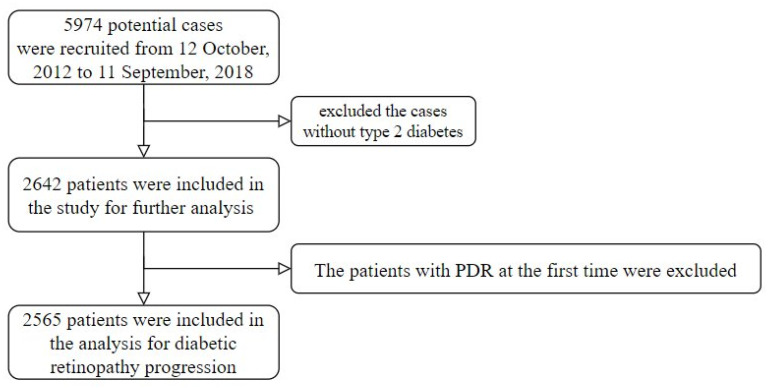
Flowchart of the enrolled patients.

A tertiary referral medical center in northern Taiwan provided the research data from 12 October 2012 to 11 September 2018. This study was approved by the Ethics Committee of Tri-Service General Hospital (IRB NO 2-105-05-073). Diabetic patients with more than 2 fundus color photography tests were included in this study. We defined time 0 (the beginning of the follow-up period) as the first eye examination. Initially, there were 5974 potential cases. However, those without type 2 DM were excluded. In this study, type 2 DM is defined as having a prescription for insulin or an oral antidiabetic and 1 of the following conditions: (1) at least two International Classification of Diseases (ICD) codes of type 2 DM (ICD9: 250 and ICD10: E11) at least 6 months from the start of the study; (2) at least 2 records of ≥126 mg/dL of blood glucose before meals (ante cibum, AC) at least 6 months from the start of the study; and (3) at least 2 records of ≥6.5% HbA1c at least 6 months from the start of the study. Moreover, patients without HbA1c and glucose AC results within 14 days before the start of the study were also excluded. Ultimately, 2642 patients with 14,409 test results were included for analysis (Figure 1).

### 2.2. Measurements and Variables

DR was graded based on the International Clinical Diabetic Retinopathy Disease Severity Scale [14] as follows: (0) no apparent retinopathy; (1) mild non-proliferative DR (NPDR): microaneurysms only; (2) moderate NPDR: any finding of microaneurysms, dot and blot hemorrhages, hard exudates, or cotton wool spots but less than severe NPDR; (3) severe NPDR: intraretinal hemorrhages (≥20 in each of the 4 quadrants), definite venous beading in 2 quadrants, or intraretinal microvascular abnormalities in 1 quadrant but no signs of proliferative retinopathy; and (4) proliferative DR (PDR): 1 or more findings of neovascularization and vitreous or preretinal hemorrhages. The end of the follow-up period was determined by the following: (1) the change in the grade of DR or (2) the end of the last fundus color photography if there was no progression of DR. The patients with PDR at the start of the study were excluded, leaving 2565 patients to be further analyzed.

The expected glucose AC was calculated by the following equation: (28.7 × HbA1c) − 46.7 [15]. The difference between the observed and expected glucose AC was referred to as the glycemic gap in this study. If there were several glucose AC tests within 14 days before the start of the study, the latest result was selected. To further explore the effect of the amplitude of glycemic excursion poor control and excessive control, we defined 2 additional variables as the following: (1) positive glycemic gap—the maximum of a series of glycemic gap within 14 days, and the negative number was defined as 0 in this calculation; (2) negative glycemic gap—the minimum of a series of glycemic gap within 14 days, and the positive number was defined as 0 in this calculation. We also collected other laboratory data from our electronic health records within 30 days before the start of the study: total cholesterol, low-density lipoprotein (LDL), high-density lipoprotein (HDL), triglyceride, creatinine, uric acid, hemoglobin, white blood cell (WBC), platelet count, neutrophils, lymphocytes, albumin, and total bilirubin. The complete blood count was obtained using the automated XN-9000 hematology analyzer (Sysmex Co., Kobe, Japan). HbA1c was tested by the automated Glycated Hemoglobin Analyzer, HLC-723 G11 (Tosoh Bioscience Inc., Tokyo, Japan). An AU5800 Series Clinical Chemistry Analyzer (Beckman Coulter Inc., Brea, CA, USA) analysis system was used to detect chemistry assays. The missing rate of mentioned laboratory data was less than 30%. We used multiple imputation to impute the missing values.

The other demographic characteristics and comorbidities were collected from our electronic health records. The collected patients’ characteristics included their gender, age, systolic blood pressure (SBP), and diastolic blood pressure (DBP). Comorbidities, including hypertension, lipidemia, ischemic heart disease, heart failure, chronic obstructive pulmonary disease (COPD), stroke, and diabetic neuropathy, were recorded after a subsequent chart review according to the ICD 9 and ICD 10 codes.

### 2.3. Deep Learning Model for Grading DR

Due to the numerous fundus color photography results, it was not possible to record them one by one. Therefore, we used a software we have developed previously to grade DR [16]. The deep learning model was based on the convolutional neural network. Kaggle, a coding website [17] with 35,126 images with corresponding DR grade, provided fundus color photography for the deep learning model. The model architecture was based on a 50-layer SE-ResNeXt [18]. The public score was 0.837 in a test with 53,576 images, which was seventh on the leaderboard, while the private score was 0.841, which was third. This model was applied to our images. Each test was conducted in both eyes, and the severity was determined by the result of the eye with more severe DR.

### 2.4. Statistical Methods

We presented the characteristics as means and standard deviations, numbers of patients, or percentages, where appropriate. They were then compared using either analysis of variance or the chi-squared test, as appropriate. A *p*-value < 0.05 was considered significant. Statistical analyses were conducted using R software version 3.4.3.

The primary analysis evaluated the effect of the different types of glycemic gaps in DR progression. Kaplan–Meier curves were obtained to evaluate the progression between the participants with higher glycemic gaps and those with lower glycemic gaps. The log-rank test was used to test the statistical significance, and the cutoff value of the glycemic gap was decided by the median. All variables were evaluated on their effect on DR progression using the univariate Cox proportional hazards model. We used the multivariable Cox proportional hazards model to adjust the potential confounding factors. The selection of the adjusted variables was based on the significance of the univariate analysis. Because the proportional hazards assumption of the Cox proportional hazards model might have been violated in our data, we also used the time-dependent receiver operating characteristic curve to present the effect of the different types of glycemic gaps with respect to time.

We performed secondary analyses with stratified analysis. Because the initial conditions of the different patients varied in real-world clinical practice, the initial grade of DR and the baseline HbA1c were used as the stratified variables. We only presented the analysis of the most significant type of glycemic gap in DR progression. The Cox proportional hazards model was used for the interaction analysis, and the adjusted variables were the same with the primary analysis.

## 3. Results

Table 1 shows the baseline characteristics of 2565 patients according to DR. In total, 1046 patients were classified as no DR, 480 patients as mild NPDR, 757 patients as moderate NPDR, and 282 patients as severe NPDR. The DR severity at the beginning of our study was significantly associated with younger age, higher creatinine level, higher HbA1c, lower albumin levels, higher uric acid levels, and more hypertensive events.

The conditional inference tree is defined as a method to minimize the information entropy from the classification of the characteristics used. Thus, it also can be used to find the most significant cutoff value of interesting predictors. We used a conditional inference tree to predict the DR progression using the glycemic gaps. Finally, an optimal cutoff value of 45 mg/dL was determined to minimize the information entropy.

Considering the baseline glycemic control, we divided the patients into three groups equally based on HbA1c level for the subgroup analysis based on the HbA1c level. Table 2 presents the basic characteristics of the patients with different glycemic controls (by HbA1c). The subjects were divided into Q1 (HbA1c < 6.8%), Q2 (HbA1c = 6.8–8.3%), and Q3 (HbA1c > 8.4%). Poorer glycemic control (higher HbA1c) was related to the DR severity, younger age, lower albumin level, and higher uric acid level.

Table 3 presents the risk of DR progression through the end of the study. The initial DR grade, glycemic control, glycemic gap, and negative glycemic gap were found to have statistical significance in HR (*p* < 0.05), both in the crude and adjusted model. The cut points of glycemic gap (45 mg/dL) and negative glycemic gap (45 mg/dL) were associated with significantly higher HR for DR progression when compared with a gap of <45 mg/dL, both in the crude and adjusted model.

**Figure 2:**
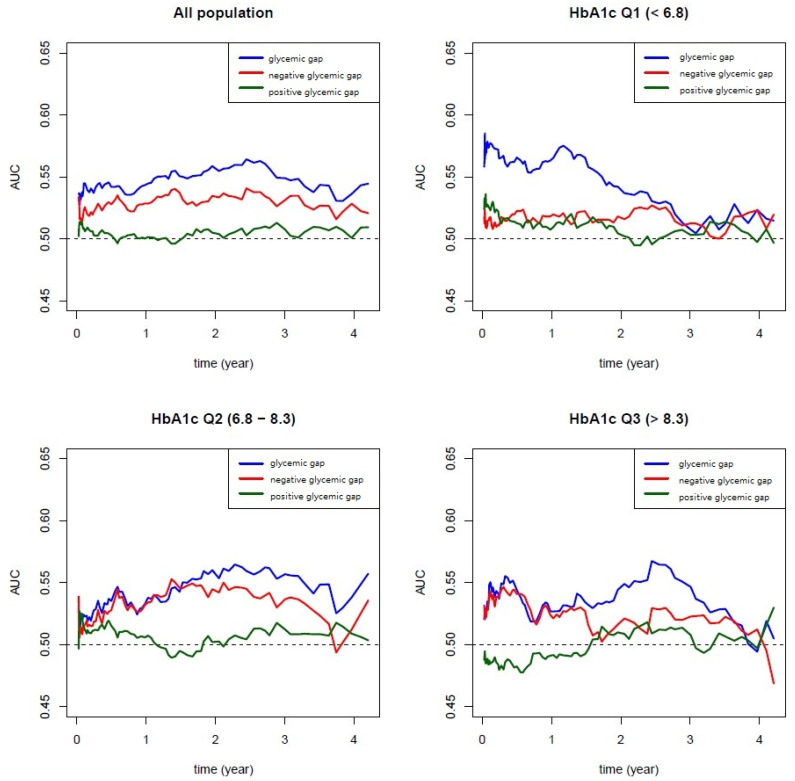
Time-dependent area under the curve (AUC): a similar association, in that the glycemic gap and negative glycemic gap both showed higher AUC for predicting diabetic retinopathy progression as compared to the positive glycemic gap, was found in all populations and each glycemic control group.

Figure 2 presents the time-dependent area under the curve (AUC). Compared with the positive glycemic gap, both the glycemic gap and negative glycemic gap showed large AUC values for predicting DR progression (Figure 2). This relationship (similar AUC association: glycemic gap > negative glycemic gap > positive glycemic gap) was found at almost every time point in all groups and in every HbA1c subgroup.

**Figure 3:**
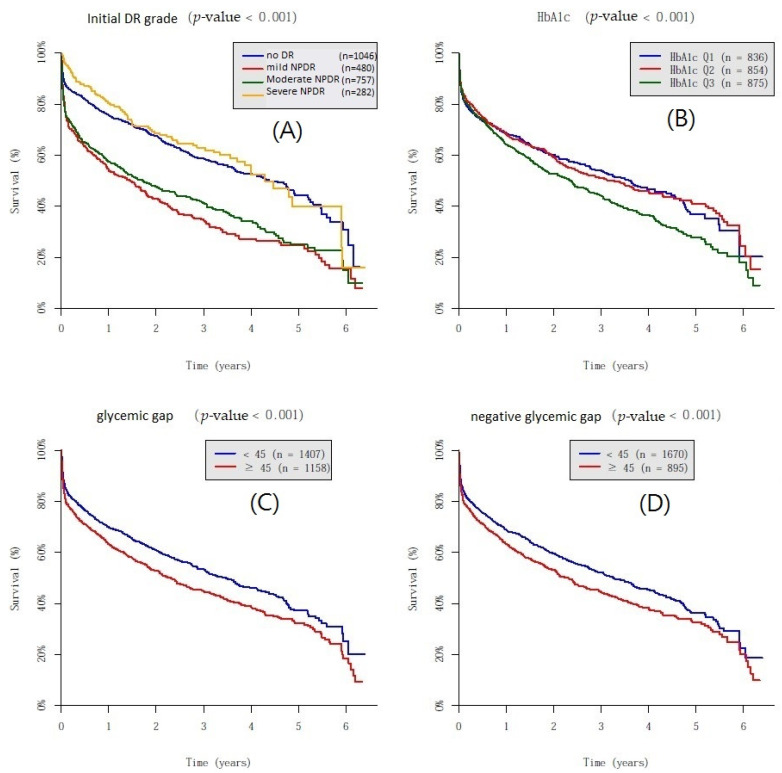
Kaplan–Meier survival curves. (**A**): Stepwise diabetic retinopathy progression with different initial grades of diabetic retinopathy. (**B**): A stepwise fashion of diabetic retinopathy progression with differing glycemic control. (**C**): A stepwise fashion of diabetic retinopathy progression with a glycemic gap > 45 mg/dL as a cutoff value. (**D**): A stepwise fashion of diabetic retinopathy progression with a negative glycemic gap > 45 mg/dL as a cutoff value.

As shown in Figure 3, the Kaplan–Meier survival curve showed that the poor glycemic control group was more positively associated with DR progression as compared with the good and intermediate glycemic control groups. In addition, the survival curve did not reveal a difference between the good glycemic control and intermediate control groups. The Kaplan–Meier survival curve showed that a glycemic gap > 45 mg/dL was associated with a significantly higher risk for DR progression when compared with a gap of <45 mg/dL for both the glycemic gap and negative glycemic gap (log-rank test *p* < 0.05).

As shown in Table 4 and Table 5, subgroup analyses were performed to determine the potential moderating effect of glycemic control and initial DR grade on the relationships between the glycemic gap and DR progression. No significant values (*p*-value for interaction < 0.05) were noted in any of the subgroups. The relationships were determined to be stronger in the no DR, severe NPDR, and HbA1c Q2 groups.

## 4. Discussion

The two major findings of the present study are as follows:

(1) The glycemic gap, by eliminating the possible influences of chronic blood glucose controls, supports the possible deteriorating effects of glucose fluctuation on the progression of DR.

(2) Both the positive and negative glucose gaps were associated with the progression of DR. Furthermore, a negative glucose gap revealed stronger AUC values in the progression of DR. The negative glycemic gap correlated with the progression of DR and indicated the negative effects of hypoglycemia episodes on the progression of DR.

To the best of our knowledge, this is the first study to examine the link between the glycemic gap and DR in type 2 DM patients. In addition, the glycemic gap may be a useful and simple tool for the assessment of GV. We suggest that the glycemic gap could be further studied as an assessment method for GV.

HbA1c has been well accepted as the gold standard for the evaluation of glycemic control, and an improvement in the HbA1c greatly reduces the risks of complications in patients with type 2 DM [19]. HbA1c-centered studies with aggressive treatment goals, such as ACCORD and VADT, have been performed. However, very low and high mean HbA1c values were associated with increased mortality in patients, revealing that tight glucose control did not improve healthcare outcomes [3,4]. Therefore, a reasonable A1C goal for adults is <7%; meanwhile, a lower glycemic goal of <7.5% for older adults was suggested in the practical guidelines [20]. This result was also true in our study. The survival curve showed that the poor glycemic control group had a significantly more rapid progression of DR, while the curve of the good glycemic control group was similar to that of the intermediate glycemic control group. Because the HbA1c only provides an average glucose level in the previous 3 months and does not reflect individual patterns of glycemic control, the development of diabetic complications is influenced not only by HbA1c but also by other factors.

In recent years, glucose fluctuation, which is also called as GV, was reported as a contributor to the development of diabetes-related complications irrespective of the HbA1c level [21,22]. DR is one of the major microvascular complications of DM. To date, numerous studies have reported that GV may also contribute to the development and progression of DR. These measurements of GV include HbA1c variability, CGM, and FPG variability.

Studies have provided evidence that long-term HbA1c variability predicted the risk of microvascular disease and DR in patients with type 2 DM [23,24,25]. In addition, the HbA1c variability was associated with macrovascular but not with microvascular diseases in type 2 DM patients, whereas short-term GV was associated with both macrovascular and microvascular events in the ADVANCE trial [26]. The HbA1c variability was associated with nephropathy but not with retinopathy in the RIACE Italian Multicenter Study [27]. One meta-analysis reported that DR was associated with the long-term HbA1c variability in type 1 DM but not in type 2 DM [28]. However, recent study with a long follow-up period reported that HbA1c variability played an important role in the development of DR when the HbA1c variability was high (>0.75% [10]; the cutoff value of HbA1c SD was 1.24 [9]). One longitudinal study in Taiwan that included 3152 type 2 DM patients who were followed-up for at least 2 years showed that HbA1c variability is an independent risk factor for DR. HbA1c variability may play a role in DR development when the mean value of HbA1c variability index is >0.75% [10]. The HbA1c standard deviation was associated with a risk of ≥3 steps of progression on the Early Treatment DR Study person scale as well as progression to PDR in a Korean retrospective study that had a follow-up period of more than 5 years [9]. The optimal cutoff value of the HbA1c SD was 1.24 for PDR and ≥3 steps of progression. GV may play a greater role in DR development when the magnitude of variation over a long period is large.

A Chinese cross-sectional study that assessed the 72 h CGM of 3262 type 2 DM patients found that the time in range (the percentage of time that the glucose range was within 70–180 mg/dL) and GV were associated with the prevalence of all stages of DR after adjusting for clinical risk factors [5,6]. Another Italian study that assessed the 72 h CGM reported that some parameters of GV were associated with DR, but its significance was lost in the multivariate analysis [29]. However, 33 type 1 diabetes mellitus and 35 type 2 diabetes mellitus cases were combined in the analysis to increase statistical power, thereby resulting in the heterogeneity of participants in the study. On the other hand, a meta-analysis revealed that the FPG variability is significantly associated with an increased risk of DR and all-cause mortality in patients with type 2 DM [8].

These studies have demonstrated that the deleterious effects of GV on DR have been underestimated. The parameters of GV have been identified to be important for the selection of the optimal treatment strategy and for the evaluation of the risk of DR. However, the studies on GV mostly measured the value of dispersion without positive and negative directionality. We could not determine whether the association resulted from hyperglycemia with poor control or iatrogenic hypoglycemia due to an intensive HbA1c treatment goal. Further research should be performed to validate whether an HbA1c with a steady GV is a better treatment goal.

The glycemic gap was found to be a good parameter for SIH. In addition, it was revealed as a good predictor for disease severity and adverse clinical outcomes in diabetic patients with liver abscesses, pneumonia, acute myocardial infarction, acute ischemic stroke, chronic obstructive lung disease, necrotizing fasciitis, and in-hospital cardiac arrest [11,12,13]. The glycemic gap cutoff values of 45.26 [30], 42 [12], and 40 [31] were reported to predict adverse outcomes simultaneously. We then used the glycemic gap as a measurement for GV, and we also calculated the positive magnitude (positive glycemic gap) and negative magnitude (negative glycemic gap).

Compared with HbA1c variation, self-monitoring of blood glucose, or CGM, the glycemic gap is a simple and reliable measurement for GV in our study, and it can be performed by drawing blood only once. We found that both the glycemic gap and negative glycemic gap have AUC values that can significantly predict the progression of DR after adjustments; this was not true with the positive glycemic gap (Figure 2). In addition, greater AUC values were found in almost any time period despite dividing the patients into three subgroups based on the HbA1c level. This suggests that GV was independently related to DR regardless of the group. We have also found that a glycemic gap greater than the cutoff level of 45 mg/dL was associated with DR progression.

High GV increases the episodes of hypoglycemia. Hypoglycemia stimulates oxidative stress, endothelial dysfunction, and inflammatory reactions associated with microvascular changes [32,33,34]. Retinal Müller cells, which are identified as the major glial component of the retina, are closely associated with astrocytes, endothelial cells, and neurons, and play a part in the regulation of the blood–retinal barrier. In DM, it has been well established that Müller cells become activated [35,36,37]. An in vitro study of rats showed that the response of retinal Müller cells to glucose excursions was different under basal and high glucose concentrations. When the glucose concentration was normal, significant glial activation was detected not only in response to a constantly high glucose level but also to alternating low and high glucose levels. When the glucose concentration was high, a response was observed only upon exposure to a lower glucose concentration. This showed that the activation of retinal Müller cells occurs in response to glucose excursions [38]. This might explain the AUC values for predicting DR progression, as shown in Figure 3. This association was also supported by previous studies showing iatrogenic hypoglycemia due to low but oscillating HbA1c levels as contributing to the risk of DR progression [39].

Our study finding suggests that beyond the average glucose level, GV has different roles in the development of DR in type 2 DM. The worsening of DR might be affected by the episodes of hypoglycemia and sudden glycemic compensation caused by treatment [40]. A negative glycemic gap correlated with the progression of DR, which supported the possible deteriorating effects of hypoglycemic episodes on the progression of DR. In clinical practice, individualized treatments with an optimal balance between the mean HbA1c and GV are important to minimize the development of diabetes complications. An individualized treatment with an achievable goal of glycemic control and maintained GV may be similar to, or even better than, intensive goals leading to glycemic fluctuations. Future longitudinal trials and analysis with similar HbA1c levels and different GV will help validate and elucidate this effect.

The strengths of our study include the use of a longitudinal design, rather than a cross-sectional design, and a well-validated grading of DR. We also collected detailed glucose records while each patient’s fundus color photography was being performed.

A few limitations of our study should be recognized. First, all patients were from a single center; thus, sampling bias and selection bias are deemed inevitable. Second, due to the longitudinal design, the results could not be used to establish a cause–effect relationship. Third, the duration of DM in each patient, which was common risk factor for DR prevalence, was not mentioned in our study. Finally, the study population were from Taiwan; we cannot assure the generalizability of the results to other populations.

## 5. Conclusions

In conclusion, the glycemic gap, as a measurement for GV, is associated with DR progression in type 2 DM patients. The association between a negative glycemic gap and progression of DR indicated the negative effects of iatrogenic hypoglycemia. Our study results suggested that individual treatment goals with a reasonable HbA1c and stable GV may help to prevent DR. An achievable goal of glycemic control with a maintained GV may be better than intensive goals with wide glycemic fluctuations. Additional randomized prospective follow-up trials could help find an optimal balance between the mean HbA1c and GV.

## Figures and Tables

**Table 1:** The baseline characteristics of patients according to diabetic retinopathy.

	Initial Grade of DR				*p*-Value
	No DR *n* = 1046	Mild NPDR *n* = 480	Moderate NPDR *n* = 757	Severe NPDR *n* = 282	
Basic characteristics					
Gender					0.201
Female	506(48.4%)	215(44.8%)	329(43.5%)	129(45.7%)	
Male	540(51.6%)	265(55.2%)	428(56.5%)	153(54.3%)	
Age (years)	63.76 ± 12.99	63.07 ± 12.26	59.59 ± 11.11	57.15 ± 11.71	<0.001
SBP (mmHg)	139.06 ± 20.30	140.37 ± 23.46	141.14 ± 21.70	141.83 ± 23.19	0.116
DBP (mmHg)	78.84 ± 11.98	78.66 ± 11.73	79.87 ± 12.21	80.62 ± 12.25	0.046
Comorbidity					
Hypertension	389(37.2%)	211(44.0%)	319(42.1%)	128(45.4%)	0.014
Lipidemia	318(30.4%)	140(29.2%)	225(29.7%)	87(30.9%)	0.948
Ischemic heart disease	222(21.2%)	125(26.0%)	169(22.3%)	69(24.5%)	0.180
Heart failure	54(5.2%)	34(7.1%)	67(8.9%)	30(10.6%)	0.002
COPD	38(3.6%)	21(4.4%)	12(1.6%)	2(0.7%)	0.001
Stroke	145(13.9%)	66(13.8%)	90(11.9%)	36(12.8%)	0.635
Diabetic neuropathy	67(6.4%)	29(6.0%)	75(9.9%)	19(6.7%)	0.019
Laboratory test					
HbA1c (%)	7.63 ± 1.91	7.91 ± 1.91	8.20 ± 2.03	8.40 ± 2.16	<0.001
Triglyceride (mg/dL)	152.89 ± 104.63	154.97 ± 118.99	158.71 ± 118.46	151.34 ± 88.38	0.671
Total cholesterol (mg/dL)	170.82 ± 40.39	167.83 ± 38.99	173.07 ± 43.54	172.47 ± 44.15	0.169
LDL cholesterol (mg/dL)	99.07 ± 33.42	98.40 ± 31.41	100.16 ± 35.22	100.33 ± 34.48	0.771
HDL cholesterol (mg/dL)	46.83 ± 13.34	46.13 ± 12.95	45.99 ± 12.90	46.65 ± 13.08	0.537
Creatinine (mg/dL)	1.37 ± 1.75	1.70 ± 2.17	1.65 ± 2.00	1.63 ± 2.01	0.002
ALT	24.94 ± 27.24	24.39 ± 31.69	22.94 ± 37.79	25.10 ± 61.13	0.677
Total bilirubin	0.64 ± 0.41	0.66 ± 0.61	0.63 ± 0.53	0.59 ± 0.35	0.200
WBC	7.40 ± 5.58	7.39 ± 2.58	7.28 ± 4.72	7.86 ± 7.21	0.444
PLT	213.06 ± 76.15	216.90 ± 74.50	221.20 ± 81.48	227.85 ± 84.12	0.019
Neutrophil	63.63 ± 11.29	64.49 ± 11.30	63.81 ± 10.73	64.29 ± 11.07	0.497
Lymphocyte	27.43 ± 10.33	26.56 ± 10.38	27.03 ± 9.85	26.72 ± 9.72	0.409
Uric acid (mg/dL)	5.88 ± 1.78	6.08 ± 1.85	6.10 ± 1.89	6.32 ± 1.81	0.001
Hb (g/dL)	12.95 ± 1.95	12.70 ± 2.01	12.59 ± 2.19	12.58 ± 2.07	0.001
Albumin (g/dL)	3.91 ± 0.53	3.88 ± 0.56	3.83 ± 0.59	3.76 ± 0.61	<0.001
Result					
Glycemic gap	49.54 ± 59.55	52.82 ± 68.25	59.75 ± 74.43	65.29 ± 94.93	0.001
Positive glycemic gap	46.43 ± 52.36	47.63 ± 40.13	52.90 ± 44.44	54.184 ± 44.18	0.008
Negative glycemic gap	45.72 ± 52.23	50.06 ± 67.47	56.70 ± 73.08	62.184 ± 93.49	<0.001

Testing by Fisher’s exact test, Wilcoxon test, or Kruskal–Wallis test, respectively; SBP: systolic blood pressure; DBP: diastolic blood pressure; HbA1c: glycated hemoglobin; LDL: low-density lipoprotein; HDL: high-density lipoprotein; Hb: hemoglobin; ALT: alanine aminotransferase.

**Table 2:** The baseline characteristics of patients based on glycemic control. (divided into 3 groups equally based on HbA1c level at the time of enrollment).

	Into 3 Groups Equally Based on HbA1c Level			*p*-Value
	HbA1c Q1 (<6.8) *n* = 836	HbA1c Q2 (6.8−8.3) *n* = 854	HbA1c Q3 (>8.3) *n* = 875	
Basic characteristics				
DR severity				<0.001
No DR	394(47.1%)	377(44.1%)	275(31.4%)	
Mild NPDR	150(17.9%)	161(18.9%)	169(19.3%)	
Moderate NPDR	217(26.0%)	232(27.2%)	308(35.2%)	
Severe NPDR	75(9.0%)	84(9.8%)	123(14.1%)	
Gender				0.377
Female	368(44.0%)	398(46.6%)	413(47.2%)	
Male	468(56.0%)	456(53.4%)	462(52.8%)	
Age (years)	62.12 ± 12.45	63.62 ± 11.87	59.34 ± 12.52	<0.001
SBP (mmHg)	139.00 ± 20.39	140.85 ± 21.13	140.78 ± 23.30	0.140
DBP (mmHg)	78.44 ± 11.53	78.72 ± 11.41	80.72 ± 12.98	<0.001
Comorbidity				
Hypertension	345(41.3%)	369(43.2%)	333(38.1%)	0.088
Lipidemia	237(28.3%)	255(29.9%)	278(31.8%)	0.301
Ischemic heart disease	189(22.6%)	204(23.9%)	192(21.9%)	0.62
Heart failure	52(6.2%)	57(6.7%)	76(8.7%)	0.109
COPD	18(2.2%)	29(3.4%)	26(3.0%)	0.296
Stroke	126(15.1%)	106(12.4%)	105(12.0%)	0.127
Diabetic neuropathy	42(5.0%)	49(5.7%)	99(11.3%)	<0.001
Laboratory test				
Triglyceride (mg/dL)	132.54 ± 73.00	142.32 ± 91.21	188.31 ± 143.38	<0.001
Total cholesterol (mg/dL)	164.75 ± 39.84	166.52 ± 37.60	181.65 ± 44.64	<0.001
LDL cholesterol (mg/dL)	95.05 ± 33.14	96.30 ± 31.20	106.59 ± 35.41	<0.001
HDL cholesterol (mg/dL)	47.39 ± 13.04	47.05 ± 13.36	44.92 ± 12.81	<0.001
Creatinine (mg/dL)	1.69 ± 2.21	1.51 ± 1.84	1.43 ± 1.75	0.015
Uric acid (mg/dL)	6.07 ± 1.86	5.97 ± 1.78	6.06 ± 1.86	0.469
Hb (g/dL)	12.54 ± 2.17	12.70 ± 1.96	13.02 ± 2.00	<0.001
Albumin (g/dL)	3.87 ± 0.57	3.91 ± 0.56	3.82 ± 0.56	0.004
Result				
Glycemic gap	31.92 ± 51.45	47.39 ± 52.99	84.18 ± 88.80	<0.001
Positive glycemic gap	28.87 ± 26.18	44.48 ± 32.84	73.87 ± 61.59	<0.001
Negative glycemic gap	29.62 ± 50.92	45.44 ± 52.43	78.58 ± 83.07	<0.001

Testing by Fisher´s exact test, Wilcoxon test, or Kruskal–Wallis test, respectively; SBP: systolic blood pressure; DBP: diastolic blood pressure; HbA1c: glycated hemoglobin; LDL: low-density lipoprotein; HDL: high-density lipoprotein; Hb: hemoglobin.

**Table 3:** The risk of diabetic retinopathy progression at the end of the study.

	Crude-HR (95% CI)	*p*-Value	Adjusted-HR (95% CI)	*p*-Value
Initial DR grade		<0.001		<0.001
No DR	1.00		1.00	
Mild NPDR	2.00 (1.72–2.32)	<0.001	1.98 (1.70–2.31)	<0.001
Moderate NPDR	1.82 (1.58–2.09)	<0.001	1.78 (1.54–2.04)	<0.001
Servere NPDR	0.87 (0.69–1.09)	0.223	0.86 (0.69–1.08)	0.191
Glycemic control		0.003		0.060
HbA1c Q1 (<6.8)	1.00		1.00	
HbA1c Q2 (6.8−8.3)	1.00 (0.86–1.15)	0.976	0.97 (0.84–1.12)	0.668
HbA1c Q3 (>8.3)	1.23 (1.07–1.41)	0.004	1.13 (0.98–1.31)	0.083
Glycemic gap		<0.001		0.001
<45 mg/dL	1.00		1.00	
>45 mg/dL	1.26 (1.13–1.42)	<0.001	1.22 (1.09–1.37)	0.001
Positive glycemic gap		0.065		0.076
<45 mg/dL	1.00		1.00	
>45 mg/dL	1.21 (0.99–1.49)	0.065	1.21 (0.98–1.48)	0.076
Negative glycemic gap		0.001		0.005
<45 mg/dL	1.00		1.00	
>45 mg/dL	1.22 (1.08–1.37)	0.001	1.18 (1.05–1.33)	0.005
Gender		0.019		0.033
Female	1.00		1.00	
Male	1.15 (1.02–1.29)	0.019	1.13 (1.01–1.27)	0.033
Age	1.00 (1.00–1.00)	0.962	1.00 (1.00–1.01)	0.945
Height	1.18 (0.92–1.51)	0.184	0.96 (0.71–1.30)	0.804
Weight	0.95 (0.74–1.21)	0.669	0.88 (0.50–1.57)	0.676
Systolic blood pressure	1.00 (1.00–1.00)	0.269	1.00 (1.00–1.00)	0.639
Diastolic blood pressure	1.00 (1.00–1.01)	0.214	1.00 (1.00–1.01)	0.300
Comorbidity				
Hypertension	1.14 (1.02–1.28)	0.027	1.07 (0.95–1.21)	0.253
Lipidemia	0.97 (0.85–1.10)	0.609	0.94 (0.82–1.07)	0.335
Ischemic heart disease	1.09 (0.96–1.25)	0.184	1.03 (0.89–1.19)	0.682
Stroke	1.10 (0.93–1.30)	0.249	1.07 (0.91–1.27)	0.415
Diabetic neuropathy	1.30 (1.07–1.59)	0.009	1.21 (0.99–1.49)	0.066
Heart failure	1.16 (0.95–1.43)	0.152	1.08 (0.87–1.34)	0.472
Laboratory test				
Triglyceride	1.00 (1.00–1.00)	0.488	1.00 (1.00–1.00)	0.602
Total cholesterol	1.00 (1.00–1.00)	0.483	1.00 (1.00–1.00)	0.420
Low-density lipoprotein	1.00 (1.00–1.00)	0.488	1.00 (1.00–1.00)	0.602
High-density lipoprotein	1.00 (1.00–1.00)	0.983	1.00 (1.00–1.01)	0.630
Hemoglobin	0.99 (0.97–1.02)	0.699	1.00 (0.97–1.03)	0.835

All result of adjusted-HR were adjusted by grade of diabetic retinopathy, gender, hypertension, and diabetic neuropathy. Crude-HR: crude hazard ratio; HR: hazard ratio; CI: confidence interval; HbA1c: glycated hemoglobin.

**Table 4:** The risk of diabetic retinopathy progression in each subgroup at the end of the study; a negative glycemic gap >45 mg/dL was the cutoff value.

		Crude-HR (95% CI)	*p*-Value	Adjusted-HR (95% CI)	*p*-Value
Initial DR grade			0.164 (interaction)		0.129 (interaction)
No DR	<45 mg/dL	1.00		1.00	
	>45 mg/dL	1.30 (1.06–1.59)	0.012	1.30 (1.06–1.59)	0.013
Mild NPDR	<45 mg/dL	1.00		1.00	
	>45 mg/dL	1.03 (0.81–1.32)	0.790	1.01 (0.79–1.29)	0.952
Moderate NPDR	<45 mg/dL	1.00		1.00	
	>45 mg/dL	1.14 (0.93–1.40)	0.200	1.14 (0.93–1.39)	0.221
Servere NPDR	<45 mg/dL	1.00		1.00	
	>45 mg/dL	1.71 (1.13–2.57)	0.010	1.76 (1.16–2.66)	0.007
Glycemic control			0.709 (interaction)		0.686 (interaction)
HbA1c Q1 (<6.8)	<45 mg/dL	1.00		1.00	
	>45 mg/dL	1.25 (0.88–1.78)	0.217	1.23 (0.87–1.76)	0.244
HbA1c Q2(6.8−8.3)	<45 mg/dL	1.00		1.00	
	>45 mg/dL	1.22 (1.00–1.50)	0.053	1.21 (0.98–1.48)	0.073
HbA1c Q3 (>8.3)	<45 mg/dL	1.00		1.00	
	>45 mg/dL	1.11 (0.92–1.34)	0.296	1.10 (0.91–1.33)	0.339

All results of the adjusted-HRs were adjusted by grade of diabetic retinopathy, gender, hypertension, and diabetic neuropathy. Crude-HR: crude hazard ratio; HR: hazard ratio; CI: confidence interval.

**Table 5:** The risk of diabetic retinopathy progression in each subgroup at the end of the study; a glycemic gap > 45 mg/dL was the cutoff value.

		Crude-HR (95% CI)	*p*-Value	Adjuseted-HR(95% CI)	*p*-Value
Initial DR grade			0.374 (interaction)		0.336 (interaction)
No DR	<45 mg/dL	1.00		1.00	
	>45 mg/dL	1.28 (1.05–1.56)	0.014	1.26 (1.03–1.53)	0.023
Mild NPDR	<45 mg/dL	1.00		1.00	
	>45 mg/dL	1.07 (0.85–1.35)	0.565	1.06 (0.84–1.33)	0.647
Moderate NPDR	<45 mg/dL	1.00		1.00	
	>45 mg/dL	1.25 (1.03–1.53)	0.025	1.25 (1.03–1.53)	0.026
Servere NPDR	<45 mg/dL	1.00		1.00	
	>45 mg/dL	1.56 (1.03–2.36)	0.036	1.56 (1.02–2.36)	0.038
Glycemic control			0.787 (interaction)		0.806 (interaction)
HbA1c Q1 (<6.8)	<45 mg/dL	1.00		1.00	
	>45 mg/dL	1.15 (0.89–1.49)	0.277	1.15 (0.89–1.48)	0.300
HbA1c Q2(6.8−8.3)	<45 mg/dL	1.00		1.00	
	>45 mg/dL	1.28 (1.05–1.57)	0.016	1.26 (1.03–1.54)	0.025
HbA1c Q3 (>8.3)	<45 mg/dL	1.00		1.00	
	>45 mg/dL	1.20 (0.98–1.48)	0.079	1.19 (0.97–1.47)	0.099

All results of adjusted-HRs were adjusted by grade of diabetic retinopathy, gender, hypertension, and diabetic neuropathy. Crude-HR: crude hazard ratio; HR: hazard ratio; CI: confidence interval.

## Data Availability

Data available in a publicly accessible repository that does not issue DOIs. The data presented in this study are openly available in kaggle; reference number [17].
